# Leptin Intake at Physiological Doses Throughout Lactation in Male Wistar Rats Normalizes the Decreased Density of Tyrosine Hydroxylase-Immunoreactive Fibers in the Stomach Caused by Mild Gestational Calorie Restriction

**DOI:** 10.3389/fphys.2018.00256

**Published:** 2018-03-21

**Authors:** Nara Szostaczuk, Juana Sánchez, Jadwiga Konieczna, Andreu Palou, Catalina Picó

**Affiliations:** ^1^Laboratory of Molecular Biology, Nutrition and Biotechnology (Nutrigenomics and Obesity), University of the Balearic Islands, Palma, Spain; ^2^CIBER de Fisiopatología de la Obesidad y Nutrición (CIBEROBN), Palma, Spain; ^3^Instituto de Investigación Sanitaria Illes Balears (IdISBa), Palma, Spain

**Keywords:** leptin supplementation, stomach, metabolic programming, leptin/ghrelin ratio, sympathetic control

## Abstract

**Introduction:** Gestational under nutrition in rats has been shown to decrease expression of sympathetic innervation markers in peripheral tissues of offspring, including the stomach. This has been linked to lower gastric secretion and decreased circulating levels of ghrelin. Considering the critical role of leptin intake during lactation in preventing obesity and reversing adverse developmental programming effects, we aimed to find out whether leptin supplementation may reverse the above mentioned alterations caused by mild gestational calorie restriction.

**Methods:** Three groups of male rats were studied at a juvenile age (25 days old) and during adulthood (3 and 6 months old): the offspring of *ad libitum* fed dams (controls), the offspring of dams that were diet restricted (20%) from days 1 to 12 of gestation (CR), and CR rats supplemented with a daily oral dose of leptin (equivalent to 5 times the average amount they could receive each day from maternal milk) throughout lactation (CR-Leptin). The density of TyrOH-immunoreactive (TyrOH^+^) fibers and the levels of Tyrosine hydroxylase (TyrOH)—used as potential markers of functional sympathetic innervation—were measured in stomach. Plasma leptin and ghrelin levels were also determined.

**Results:** Twenty five-day-old CR rats, but not CR-Leptin rats, displayed lower density of TyrOH^+^ fibers (−46%) and TyrOH levels (−47%) in stomach compared to controls. Alterations in CR animals were mitigated at 6 months of age, and differences were not significant. Adult CR-Leptin animals showed higher plasma ghrelin levels than CR animals, particularly at 3 months (+16%), and a lower leptin/ghrelin ratio (−28 and −37% at 3 and 6 months, respectively).

**Conclusion:** Leptin intake during lactation is able to reverse the alterations in the density of TyrOH^+^ fibers in the stomach and normalize the increased leptin/ghrelin ratio linked to a mild gestational calorie restriction in rats, supporting the relevance of leptin as an essential nutrient during lactation.

## Introduction

An adverse perinatal environment is known to negatively affect the health of offspring in the long term (Godfrey and Barker, 2001; Gluckman et al., 2005; Picó et al., 2012). In this regard, there is epidemiological evidence linking under nutrition during fetal life with low birth weight and the major propensity to obesity and metabolic syndrome in adulthood (Ravelli et al., 1976; Godfrey and Barker, 2001; Gluckman et al., 2005). Several animal studies have also explored the effects of maternal under nutrition during gestation and have found that even a mild calorie restriction during the first period of gestation exerts detrimental effects in the offspring regarding energy balance and metabolic regulation, thus increasing the susceptibility to develop obesity-related pathologies in adulthood, especially when exposed to dietary stressful conditions (Vickers et al., 2000; Palou et al., 2010). These programming effects and their potential reversion require special attention, particularly when considering strategies for obesity prevention from the early stages of life.

These detrimental effects have been attributed to an impairment in insulin and leptin sensitivity (Palou et al., 2012), together with defects in hypothalamic structure and function (Garcia et al., 2010), and alterations in markers of sympathetic innervation of white and brown adipose tissues (Garcia et al., 2011; Palou et al., 2015) and of stomach (Garcia et al., 2013), leading to a higher risk to develop hallmark features of the metabolic syndrome.

The decreased expression of Tyrosine hydroxylase (TyrOH), used as indicator of sympathetic innervation, in the stomach of pups submitted to mild calorie restriction during gestation was found to be associated with lower blood ghrelin levels (Garcia et al., 2013). It is known that gastric sympathetic activation increases gastric ghrelin secretion, and noradrenaline released from sympathetic nerve terminals has been postulated as the principal factor involved in this process (Spencer et al., 2015). Ghrelin, together with leptin, are important appetite-regulating hormones. Unlike leptin, ghrelin, is known to enhance appetite and increase food intake (Wren et al., 2001). However, circulating ghrelin has been reported to be decreased in obese subjects and inversely correlated with body mass index (Tschop et al., 2001). Moreover, the prevalence of insulin resistance and type 2 diabetes is associated with low ghrelin concentrations (Poykko et al., 2003). In turn, a low leptin/ghrelin (L/G) ratio has been considered as a clinical biomarker for predicting better metabolic adaptation and increased weight loss after dietary intervention in obese women (Labayen et al., 2011) and subsequent weight maintenance after intervention in obese subjects (Crujeiras et al., 2010, 2014). This ratio has been described to decrease in type 2 diabetes patients who experienced an improvement in insulin sensitivity (Hajimohammadi et al., 2017).

Leptin is a natural component of breast milk (Casabiell et al., 1997), and is considered an essential nutrient during lactation in protecting against the development of obesity and associated pathologies in adulthood (Pico et al., 2007; Sanchez et al., 2008; Palou and Pico, 2009). Leptin supplementation at physiological doses throughout lactation has been shown to reverse some of the malprogrammed effects associated to mild calorie restriction during gestation, such as alterations in hypothalamic structure and function (Konieczna et al., 2013) and adipose tissue innervation (Konieczna et al., 2015). However, whether leptin supplementation throughout lactation is able to reverse the aforementioned decrease in TyrOH expression in the stomach and its potentially related alterations associated to gestational calorie restriction has not yet been explored.

In this study we aimed to find out whether leptin supplementation throughout lactation in rats delivered from dams submitted to mild calorie restriction during gestation reverses the decreased TyrOH expression in the stomach and normalizes circulating ghrelin levels.

## Materials and methods

### Experimental design

The animal research protocol followed in the present study was approved by the Bioethical Committee of the University of the Balearic Islands (Resolution Numbers 1798 February 18th, 2009, and 8453 June 16th, 2010), and University guidelines for the care and use of laboratory animals were followed.

Animals used in this study were housed under standard conditions, namely, controlled temperature (22°C), and 12 h light-dark cycle. They were offered free access to tap water, and were fed standard normal fat chow diet (3.3 Kcal g^−1^, with 8.0% calories from fat; PanLab, Barcelona, Spain), unless specified otherwise.

#### Study 1. short-term effects of leptin treatment during lactation in the offspring of gestational calorie restricted rats

The study was performed in 25-day-old male Wistar rats divided into three experimental groups: offspring of dams fed *ad libitum* with standard chow diet during gestation (controls); offspring of dams that were diet restricted (20%) from days 1 to 12 of pregnancy (CR); and CR rats supplemented throughout lactation with a daily oral dose of leptin, which was increased progressively from 1 ng on postnatal day 1–43.8 ng on postnatal day 20 (CR-Leptin). This amount of leptin was equivalent to five times the average amount ingested normally from maternal milk (Sanchez et al., 2005). Animals were from 17 different litters (7 from control and 10 from calorie-restricted dams). The animal protocol followed to obtain the experimental groups was previously described in detail (Konieczna et al., 2013). Briefly, virgin female Wistar rats (Charles River Laboratories, Barcelona, Spain) were mated with control male Wistar rats. Day of conception (considered as day zero of pregnancy) was determined by the presence of sperm in vaginal smears. Once pregnant, the rats were individually housed and separated into two groups: control dams, fed *ad libitum* with standard chow diet, and calorie-restricted dams (CR-dams), subjected to a 20% calorie restriction from day 1 to 12 of gestation. Calorie restriction was performed as previously described (Palou et al., 2010). After this period, all dams were fed *ad libitum*. After delivery (day 1) excess pups were removed from each litter to maintain 10 pups per dam. No differences were found in the size of the litter between controls and CR dams, nor in the male to female ratio. CR-dam pups were randomly distributed into two groups: CR and CR-Leptin. Throughout lactation, CR-Leptin pups were treated each day with an oral solution of recombinant murine leptin (PeproTech, London, UK) dissolved in water, using a pipette. The daily doses (ng) for the consecutive 20 days of lactation were 1.0, 2.0, 3.0, 4.0, 5.0, 6.3, 7.5, 8.8, 10.0, 11.3, 15.6, 17.2, 18.8, 20.3, 21.9, 23.5, 25.0, 26.6, 39.4, and 43.8 (Pico et al., 2007). Control and CR pups were given the same volume of the vehicle (water).

Pups were weaned at postnatal day 21, and then 18 male pups from the control group, 17 from the CR group, and 17 from the CR-Leptin group were housed in groups of two animals per cage and fed with a standard diet. Body weight and food intake were followed. To calculate cumulative food intake, we recorded the amount of food consumed by the two animals located in each cage during the whole experimental period. The data obtained for each cage was divided by two to estimate the intake of each individual rat, which was used to calculate the average of the cumulative food intake for each group (control, CR and CR-Leptin). Body fat content was measured at postnatal day 25 using quantitative magnetic resonance spectroscopy (EchoMRI-700™, Echo Medical Systems, LLC., TX, USA).

On day 25 of age, pups were killed by decapitation during the first 2 h of the beginning of the light cycle, under fed conditions. Blood samples were collected from the neck in heparinized containers. They were subsequently centrifuged at 1,000 × g for 10 min to obtain the plasma, and stored (−20°C) until analysis. Some of the animals of this cohort (*n* = 10–11 per group) were used to perform gene expression and Western blot analysis in stomach. For that, the stomach was rapidly removed, opened, and rinsed with saline containing 0.1% diethyl pyrocarbonate (Sigma, Madrid, Spain). It was then frozen in liquid nitrogen and stored at −80°C until use. The other animals of the cohort (*n* = 6–8 per group) were used for morphometric and immunohistochemical analysis in stomach.

#### Study 2. long-term effects of leptin treatment during lactation in the offspring of gestational calorie restricted rats

A second study was conducted in a different cohort of male Wistar rats to determine long-term effects of calorie restriction during gestation and of leptin treatment during lactation on markers of sympathetic innervation to the stomach. Thus, three groups of male animals (control, CR and CR-Leptin) were defined as in study 1 and followed up until the age of 6 months. Animals were from 20 different litters (6 from control and 14 from calorie-restricted dams). A maximum of two rats per group were siblings to minimize the effect of litter.

Blood samples were collected from saphenous vein under 12 h fasting conditions at 3 months and 1 week before sacrifice, at 6 months of age, in heparinized containers. Plasma was obtained as above described and stored until analysis. Body fat content was measured in all animals at 6 months of age, before their sacrifice, as described above. Animals were killed by decapitation at the age of 6 months under fed conditions, during the first 2 h of the beginning of the light cycle. The stomach of adult animals was removed, washed, weighed and divided longitudinally into two sections; one of them was scraped off with a glass slide and used to perform gene expression and Western blot analysis and the other one was used for morphometric and immunohistochemical analysis.

### Quantification of plasma and gastric ghrelin concentration

Total ghrelin concentration in plasma and stomach was measured using a rat ghrelin enzyme immunoabsorbent assay (EIA) kit (Phoenix Europe GmbH, Karlsruhe, Germany). For ghrelin determination in gastric mucosa, ghrelin peptide was extracted as previously described (Sanchez et al., 2004). Briefly, samples were homogenized at 4°C in 1:3 (w/v) PBS (137 mM NaCl, 2.7 mM KCl, and 10 mM phosphate buffer, pH 7.4) using a polytron homogenizer. The homogenates were then centrifuged (7,000 × g, for 2 min at 4°C). The supernatants were mixed with 1 M acetic acid containing 20 mM HCl (10 volumes), boiled (20 min), and centrifuged (7,000 × g for 2 min at 4°C). Finally, the supernatants were lyophilized and resuspended in PBS and used for ghrelin measurement.

### Western blot analysis for tyrosine hydroxylase

Stomach was homogenized at 4°C in 1:3 (w/v) of PBS containing Halt Protease and Phosphatase Inhibitor Cocktail (Thermo Fisher, Rockford, IL, USA) with a Polytron homogenizer (VWR). After centrifuging the homogenate (700 × g, 10 min, 4°C), the supernatant was used for total protein and TyrOH analysis. The amount of TyrOH was determined by Western blot. For this, 150 μg of total protein (measured by the Bradford method) was solubilized and boiled (3 min) in Laemmli sample buffer containing 5% 2-beta-mercaptoethanol. Proteins were then fractioned by 10% SDS-polyacrylamide gel electrophoresis, and electrotransferred onto a nitrocellulose membrane (Bio-Rad Laboratories, Madrid, Spain). After blocking, the membranes were incubated overnight with polyclonal rabbit anti-TyrOH (Catalog number: TH (H-196): sc-14007, Santa Cruz Biotechnology, Inc., CA, USA), and for 1 h with monoclonal mouse anti-β-Actin (Catalog number: β-Actin (8H10D10) Mouse mAb #3700) as loading control. Both primary antibodies were diluted 1:1,000 in TBS-T. Secondary anti-IgG antibodies used were: infrared-dyed anti-Rabbit IgG antibody, and infrared-dyed anti-Mouse IgG antibody, in both cases from LI-COR Biosciences (Lincoln, NE, USA), and diluted 1:20,000. For infrared detection, membranes were scanned using an Odyssey Infrared Imaging System, and bands were quantified using the software Odyssey V3.0 (LI-COR Biosciences).

### Real-time quantitative polymerase chain reaction (RT-qPCR) analysis

Total RNA was extracted from the stomach using the Tripure Reagent (Roche Diagnostic Gmbh, Mannheim, Germany) following the manufacturer's instructions. RNA was subsequently quantified using the NanoDrop ND-1000 spectrophotometer (NadroDrop Technologies, Wilmington, DE, USA) and agarose gel electrophoresis was used to confirm its integrity.

mRNA levels of ghrelin in stomach were measured by RT-qPCR. 0.25 μg of total RNA (in a total volume of 5 μl) was denatured (65°C for 10 min) and reverse transcribed into complementary DNA (cDNA) by using MuLV reverse transcriptase (Applied Biosystem, Madrid, Spain) at 20°C for 15 min, 42°C for 30 min, and 5 min at 95°C in an Applied Biosystems 2720 Thermal Cycler (Applied Biosystem, Madrid, Spain). Each PCR was performed from diluted cDNA template (1/5), forward and reverse primers (1 μM each), and Power SYBER Green PCR Master Mix (Applied Biosystems, CA, USA). RT-qPCR was performed using the Applied Biosystems StepOnePlus™ Real-Time PCR Systems with the following conditions: 95°C for 10 min, followed by 40 cycles with two temperatures: 95°C for 15 s and 60°C for 1 min. A melting curve was produced after each run in order to verify the purity of the products. The threshold cycle (Ct) was calculated using the StepOne Software v2.2.2, and relative gene expression was calculated as a percentage of control animals, using the 2^−ΔΔ*Ct*^ method (Livak and Schmittgen, 2001) with 18S and *Gdi-1* (GDP dissociation inhibitor 1) as reference genes. Sequences of primers were: ghrelin, forward: 5′-cagaaagcccagcagagaaa-3′, reverse: 5′-gaagggagcattgaacctga-3′; 18S, forward: 5′-cgcggttctattttgttggt-3′, reverse: 5′-agtcggcatcgtttatggtc-3′; and *Gdi-1*, forward: 5′-ccgcacaaggcaaatacatc-3′, reverse: 5′-gactctctgaaccgtcatcaa-3′. They were obtained from Sigma (Madrid, Spain).

### Morphometric and immunohistochemical analysis

Immunohistochemical analysis of TyrOH was performed in stomach samples (*n* = 6–8 animals per group at 25 days, and *n* = 5–8 animals per group at 6 months). For this purpose, samples were dehydrated in graded series of ethanol, cleared in xylene and embedded in paraffin. Five-micrometer-thick sections of tissue were cut with a microtome and mounted on slides. The immunohistochemical detection of TyrOH was performed with the avidin-biotin peroxidase (ABC) staining method (Hsu et al., 1981). Following rehydration, sections were incubated sequentially in the following solutions (at room temperature, unless specified): 3% hydrogen peroxide in H_2_O for 10 min to block endogenous peroxidase; 2% goat normal serum in PBS (Phosphate-buffered saline) (0.01 M, pH = 7.4, for 20 min); polyclonal rabbit anti-TyrOH antibody, AB1542, (Millipore Corporation, 28820 Single Oak Drive, Temecula, CA), diluted 1:200 in PBS with 1% BSA) overnight at 4°C; biotinylated goat anti-rabbit IgG (Vector Laboratories, Burlingame, CA) diluted 1:200 in PBS for 30 min; peroxidase-labeled ABC reagent (Vectastain ABC kit, Vector) in PBS for 1 h, and Fast 3,3′-diaminobezidine tablet, DAB (Sigma, St. Louis, MO, USA) in H_2_O for 3 min. Slides were subsequently washed with deionized water, then counterstained with hematoxylin, dehydrated through graded alcohols, cleared in xylene, and mounted with Eukitt (Panreac Química SA). Negative controls consisting in the omission of the primary antibody were performed. Immunoreactive TyrOH (TyrOH^+^) fibers were counted using AxioVision 40V 4.6.3.0. Software (Carl Zeiss, Imaging Solutions GmbH, Germany). The specific immunoreactive signal was indicated by an intense brown color. TyrOH^+^ fibers were counted in 2–4 different areas of the muscular layer area per animal in 25 day-old animals and in 1–3 areas (generally 3) of the inner oblique muscular layer per animal in 6 month-old animals. It was ensured that the orientation of the stomach was the same in all samples to be able to confront the data from the different animals. The number of fibers was always counted following the same procedure, starting from the left side to the right side of the selected area (Figure S1). The counting was made in duplicate and in a blinded fashion. Internal positive controls (nerves in the parenchyma of the stomach) were detected in most cases (Figure S2).

### Statistical analysis

No blinding was performed for the analysis of the data. Data are expressed as mean ± s.e.m. (standard error of the mean). For multiple comparisons, repeated measures analysis of variance (ANOVA) was done to assess the effects of different factors: perinatal treatment (controls, CR, CR-Leptin) and age of animals (3 or 6 months) in study 2. Two-way ANOVA was also used to evaluate the effects of perinatal treatment (controls, CR, CR-Leptin) and the age of animals (25 days, 6 months) on the number of TyrOH+ fibers in the stomach, considering data from studies 1 and 2. One-way ANOVA was performed to assess differences between groups (controls, CR, CR-Leptin) at the age of 25 days (study 1), and at the ages of 3 and 6 months (study 2), when no significant differences were found considering both ages together, and for data obtained only at the age of 6 months. ANOVA was followed by a least significant difference (LSD) *post-hoc* test. In addition, Student's *t*-test was used for single comparisons between groups. *P* < 0.05 was defined as the threshold of significance. Analysis was carried out with SPSS for Windows (SPSS version 24.0, Chicago, IL, USA).

## Results

### Study 1

Body weight and fat mass of animals at the age of 25 days are shown in Table 1. As described in the same cohort of animals (Konieczna et al., 2013), the offspring of calorie restricted dams during gestation showed lower body weight and body fat than controls (*P* < 0.05, LSD *post-hoc* one-way ANOVA). Leptin treatment throughout lactation had no apparent effects on the abovementioned parameters at this young age.

**Table 1:** Anthropometric, plasma and stomach parameters of animals at the age of 25 days.

	**Control**	**CR**	**CR-Leptin**	**ANOVA**
**JUVENILE AGE (25 DAYS)**				
**Anthropometric parameters**				
Body weight (g)	65.5 ± 1.1^a^	59.6 ± 1.2^b^	60.2 ± 1.2^b^	T
Fat mass (%)	7.37 ± 0.28^a^	5.56 ± 0.20^b^	5.87 ± 0.22^b^	T
**Plasma parameters**				
Leptin (μg/L)	2.21 ± 0.17^a^	1.79 ± 0.12^b^	2.04 ± 0.05^ab^	T
Ghrelin (μg/L)	2.20 ± 0.28	1.66 ± 0.30	2.26 ± 0.67	
Leptin/ghrelin	1.11 ± 0.20	0.91 ± 0.35	1.45 ± 0.66	
**Stomach parameters**				
Stomach weight (mg)	478 ± 12	513 ± 17	481 ± 18	
Ghrelin mRNA (Au)	100 ± 7	80 ± 8	76 ± 6	
Ghrelin levels (ng/g)	221 ± 24	225 ± 18	239 ± 18	

*Data are mean ± s.e.m. Circulating leptin and ghrelin were determined under feeding conditions. T, effect of early treatment (C, CR and CR-Leptin) (P < 0.05, one-way ANOVA). Symbols: a ≠ b*.

**Figure 1:**
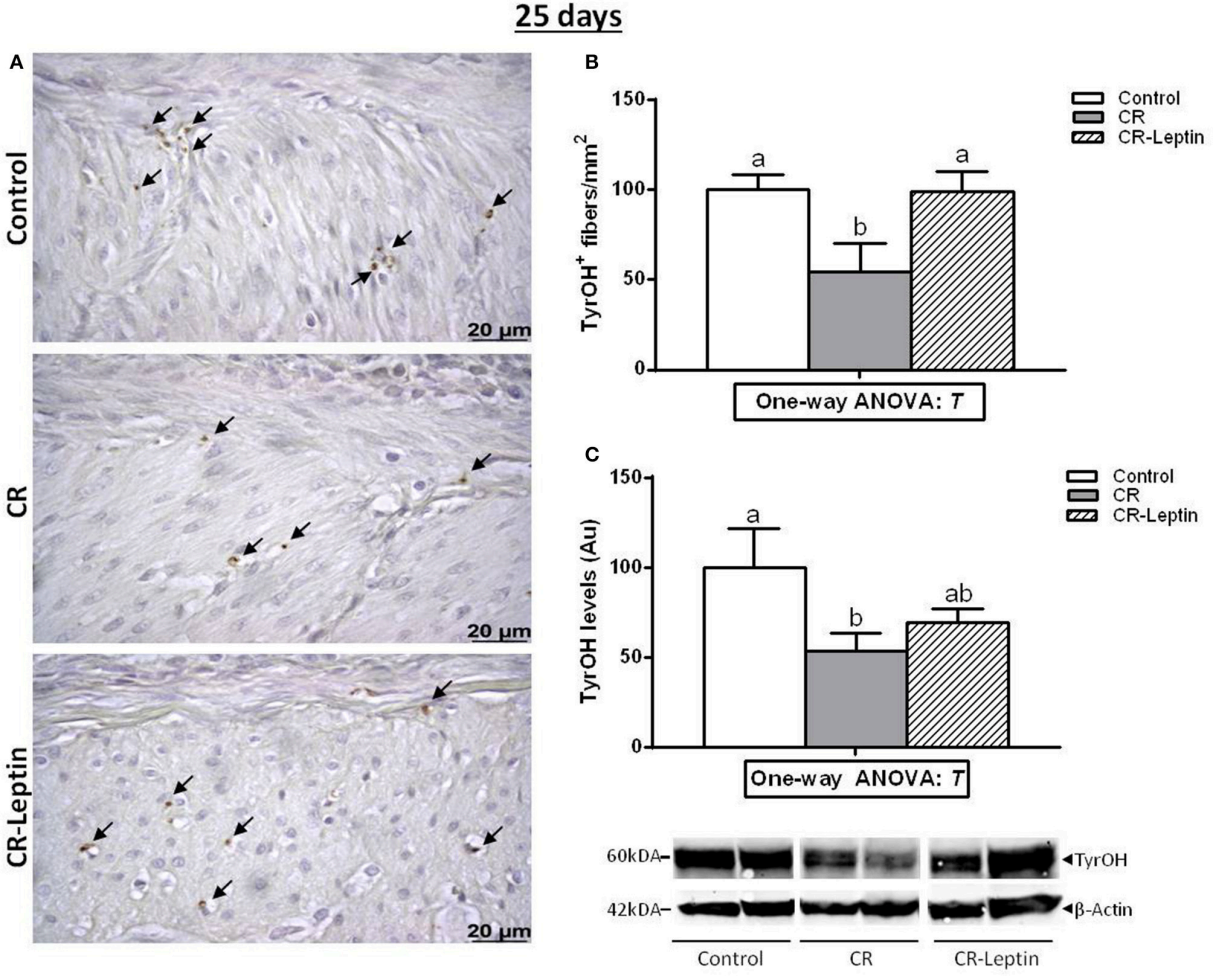
Immunohistochemical analysis of TyrOH positive (TyrOH^+^) fibers and western blot determination of specific TyrOH protein in the stomach of control, CR and CR-Leptin animals at the age of 25 days. **(A)** Representative immunostained sections for TyrOH (brown color), indicated by black head arrows in the muscular layer area. Scale bar: 20 μm. **(B)** Number of TyrOH^+^ (fibers/mm^2^ of stomach). **(C)** Specific protein levels of TyrOH in the stomach expressed relative to levels of β-Actin (loading control). Representative bands for TyrOH and β-Actin are shown. They have been cut from the same membrane and grouped together in the image. 150 ug of protein were loaded in each lane. Results are expressed as mean ± s.e.m. (*n* = 10–11). Symbols: T, effect of early treatment (C, CR, and CR-Leptin) (*P* < 0.05, one-way ANOVA). Symbols: a ≠ b.

TyrOH was assessed by using both morphological (tissue immunostaining) and biochemical (protein content) approaches. Immunohistochemical analysis of TyrOH positive (TyrOH^+^) fibers and western blot determination of specific TyrOH protein in the stomach of 25 day-old animals is shown in Figure 1. TyrOH^+^ fibers were mainly detected in the muscular layer area of the stomach. They were also detected in the submucosa, but in smaller numbers, so they were not quantified. CR animals displayed significantly lower number of TyrOH^+^ fibers in the muscular layer compared to controls (54 ± 16 vs. 100 ± 8), whereas the alteration was totally reverted in CR-Leptin animals (99 ± 11; *P* < 0.05, LSD *post-hoc* one-way ANOVA). Analysis of specific TyrOH protein by western blot also revealed a significant decrease of TyrOH levels in the stomach of CR animals compared to controls, whereas CR-Leptin animals showed intermediate levels which were not different from control and CR animals (*P* < 0.05, LSD *post-hoc* one-way ANOVA).

We therefore determined whether the decreased number of TyrOH^+^ fibers in the stomach in CR animals was associated with an alteration in circulating ghrelin levels (Table 1). In the present study, no significant differences between groups were found regarding blood ghrelin levels. No significant differences were either found regarding ghrelin expression or protein levels in the gastric mucosa, and no alterations were observed in the weight of the stomach.

Circulating leptin levels are shown in Table 1. As previously described (Konieczna et al., 2013), CR animals displayed lower plasma leptin levels than controls, while CR-Leptin animals showed intermediate levels, which were not different from controls at the level of *P* < 0.05 (LSD *post-hoc* one-way ANOVA). No significant differences were found regarding the L/G ratio.

### Study 2

A new cohort of animals was followed up until 6 months of age with the purpose of determining whether the effects of maternal calorie restriction and of leptin treatment during lactation found at a juvenile age were maintained in adulthood.

Detailed anthropometric characterization of animals from 21 days to 6 months, and blood parameters at 6 months have been previously published (Szostaczuk et al., 2017). Some of the body weight-related parameters, as well as body fat percentage at 6 months and cumulative food intake from 21 days to 6 months are included in Table 2. No differences between groups were found regarding body weight, body fat and cumulative food intake.

**Table 2:** Anthropometric characteristics, food intake, and plasma and stomach parameters of adult animals.

		**Control**		**CR**		**CR-Leptin**		**One-wayANOVA**	**RepeatedmeasuresANOVA**
**ADULTHOOD**									
**Anthropometric parameters and food intake**									
Body weight (g)	3 months	355 ± 8		370 ± 9		363 ± 7			
	6 months	442 ± 21		461 ± 20		442 ± 18			
Fat mass (%) (6 m)		16.5 ± 1.1		18.8 ± 0.9		18.2 ± 1.0			
Cumulative food intake (Kcal) (21 days to 6 months)		11, 731 ± 153		11, 326 ± 163		11, 415 ± 165			
**Plasma parameters**									
Leptin (μg/L)	3 months	1.81 ± 0.27		2.99 ± 0.46		2.84 ± 0.37			
	6 months	2.76 ± 0.34		3.78 ± 0.85		2.68 ± 0.33			
Ghrelin (μg/L)	3 months	21.3 ± 1.1^ab^		18.7 ± 0.6^a^		22.3 ± 1.0^b^		T	Age
	6 months	15.2 ± 0.7^*^		14.4 ± 0.9^*^		15.8 ± 1.4^*^			
Leptin/ghrelin	3 months	0.09 ± 0.02	^A^	0.18 ± 0.04	^B^	0.13 ± 0.02	^A^		T, Age
	6 months	0.16 ± 0.02^*^		0.27 ± 0.06		0.17 ± 0.03^*^			
**Stomach parameters (6 m)**									
Stomach weight (mg)		2, 036 ± 65		2, 194 ± 102		2, 194 ± 86			
Ghrelin mRNA (Au)		100 ± 17		115 ± 18		113 ± 25			

*Data are mean ± s.e.m. Circulating leptin and ghrelin levels were determined under fasting conditions. Repeated measures analysis of variance (ANOVA) was performed to assess the effects of perinatal treatment (controls, CR, CR-Leptin) and the age of animals (3 or 6 months). One-way ANOVA was carried out to determine differences between groups at the ages of 3 and 6 months separately, when no significant differences were found considering both ages together, and for data obtained only at the age of 6 months. ANOVA was followed by a least significant difference (LSD) post-hoc test. Student's t-test was used for single comparisons between groups. Symbols: T, effect of early treatment (C, CR and CR-Leptin); Age, effect of age (6 months vs. 3 months) (P < 0.05, Repeated measures ANOVA). Symbols: A ≠ B for repeated measures ANOVA or a ≠ b for one-way ANOVA*.

* *Different from their respective value at 3 months of age (Student's t-test)*.

**Figure 2:**
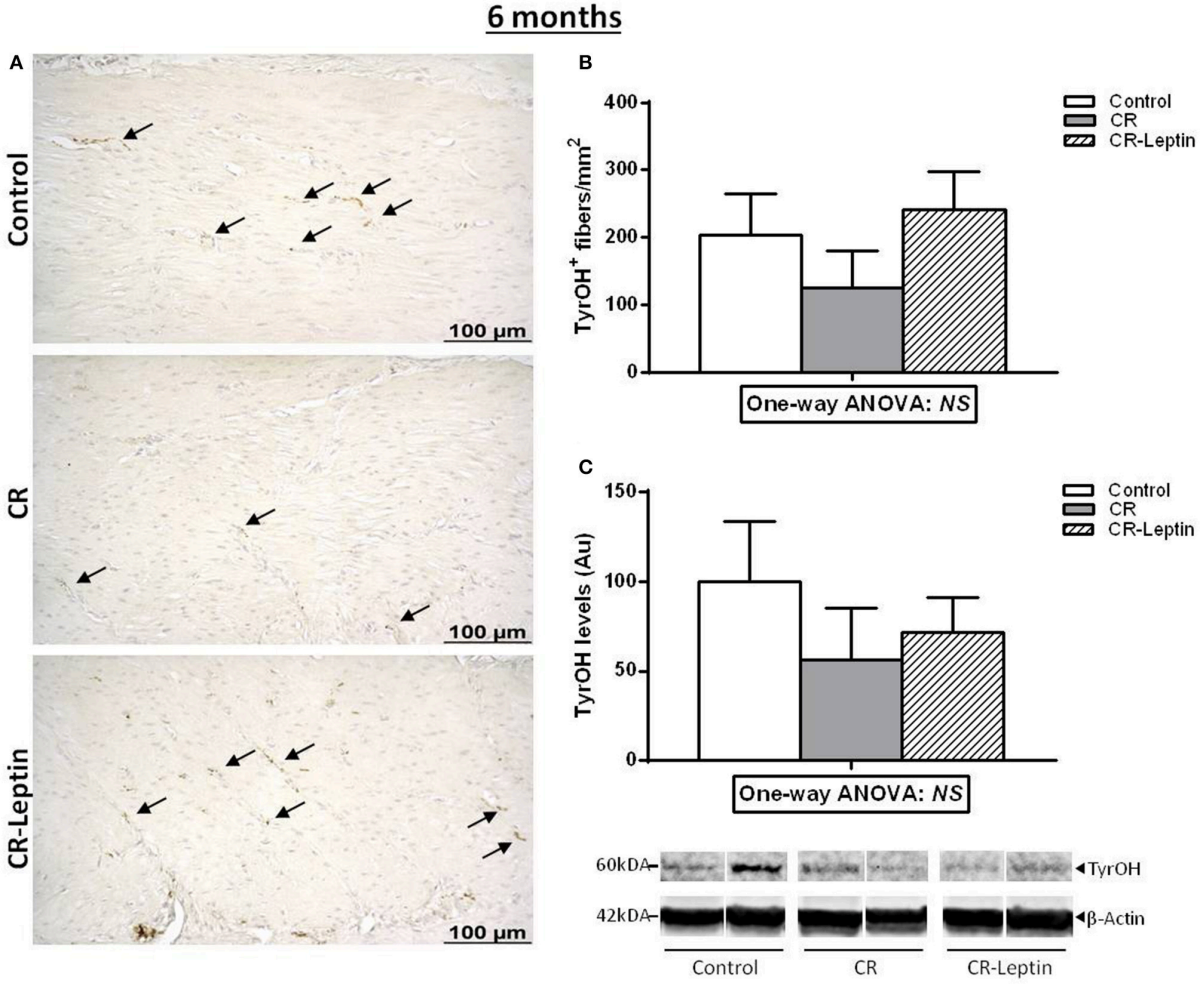
Immunohistochemical analysis of TyrOH positive (TyrOH^+^) fibers and western blot determination of specific TyrOH protein in the stomach of control, CR and CR-Leptin animals at the age of 6 months. **(A)** Representative immunostained sections for TyrOH (brown color), indicated by black head arrows, in the inner oblique muscular layer of the stomach. Scale bar: 100 μm. **(B)** Number of TyrOH^+^ (fibers/mm^2^ of stomach). **(C)** Specific protein levels of TyrOH in the stomach expressed relative to levels of β-Actin (loading control). Representative bands for TyrOH and β-Actin are shown. They have been cut from the same membrane and grouped together in the image. 150 ug of protein were loaded in each lane. Results are expressed as mean ± s.e.m. (*n* = 7–9). NS: no significant differences by one-way ANOVA.

TyrOH^+^ fibers were also determined by immunohistochemical analysis in the stomach of 6 month-old animals (Figure 2). This measurement was performed in the inner oblique muscular layer, since it was the most comparable layer between the different animals. Results showed that changes observed regarding the number of TyrOH^+^ fibers and specific TyrOH levels in the stomach between groups at a juvenile age appear to weaken in adulthood, and differences were not significant (Figure 2). Notably, when analyzing together the data of both studies (1 and 2) regarding the number of TyrOH^+^ fibers, a significant effect of the perinatal treatment was found considering both ages (25 days and 6 months) (*P* < 0.05, two-way ANOVA). *Post-hoc* analysis showed that the number of TyrOH^+^ fibers in the CR group was lower than controls (*P* < 0.05) and, than CR-Leptin animals (*P* < 0.05), while no significant differences were found between control and CR-Leptin animals considering both ages. No significant effect of age or interaction between age and treatment were found.

Regarding circulating ghrelin, at 3 months of age, CR-Leptin animals presented greater fasting ghrelin levels than CR animals, whereas control animals showed intermediate levels (*P* < 0.05, LSD *post-hoc* one-way ANOVA) (Table 2). No significant differences between groups were found at the age of 6 months. An effect of age was found considering data at 3 and 6 months: ghrelin levels at 6 months were significantly lower than at 3 months (effect of age, *P* < 0.05, repeated measures ANOVA). Similar to the results found at a juvenile age, no significant differences between groups were found concerning ghrelin expression or the weight of the stomach in 6 month-old animals.

No significant differences between groups were found regarding fasting circulating leptin levels in adult rats, either at 3 or 6 months (Table 2). However, CR animals, but not CR-Leptin animals, displayed a greater L/G ratio than controls, considering data at 3 and 6 months (effect of treatment, *P* < 0.05, repeated measures ANOVA). An effect of age was also found in the L/G ratio at 3 and 6 months in the different groups of animals. More precisely, animals at 6 months of age showed a higher L/G ratio compared to values at 3 months (effect of age, *P* < 0.05, repeated measures ANOVA).

## Discussion

Gestational calorie restriction has been reported to trigger detrimental effects in the offspring by causing a number of metabolic disturbances and affecting the capacity to properly regulate energy balance in adulthood (Vickers et al., 2000; Picó et al., 2012). Leptin supplementation at physiological doses throughout lactation has been shown to revert, at least in part, the majority of the malprogramming effects in the offspring associated to a mild calorie restriction during gestation (Konieczna et al., 2013, 2015), hence normalizing to a greater extent the phenotypic alterations observed in adulthood (Szostaczuk et al., 2017).

Among the mechanisms responsible for phenotypic alterations in the offspring of gestational calorie restricted dams, we have previously reported the presence of a decreased expression of TyrOH in the stomach (Garcia et al., 2013). TyrOH is the limiting enzyme for catecholamine synthesis and is used as a marker for sympathetic innervation (Giordano et al., 2005; Garcia et al., 2013). This alteration was proposed as the responsible cause, or at least one of the contributing factors, for the presence of reduced circulating ghrelin levels in these animals (Garcia et al., 2013), due to the involvement of sympathetic stimulation in regulating gastric ghrelin secretion (Mundinger et al., 2006; Hosoda and Kangawa, 2008). It should be mentioned that these alterations were found at early ages, before the phenotypic changes described in adult animals became evident. The present study has confirmed the presence of a reduced TyrOH content and a reduced number of TyrOH^+^ fibers in the stomach of 25 day-old pups born from dams submitted to a calorie restriction during gestation. Moreover, we present the first evidence here that this alteration was reversed in pups treated with physiological doses of oral leptin throughout lactation. Differences in TyrOH content and in the number of TyrOH^+^ fibers between groups found at a juvenile age were less marked and not significant at the age of 6 months. It must be mentioned that although the measurement of TyrOH content and TyrOH^+^ fibers in the stomach is used as a marker for sympathetic innervation (Giordano et al., 2005; Garcia et al., 2013), this method has the limitation that it does not allow measuring the number of sympathetic fibers that may not be expressing TyrOH or not at levels detectable by immunohistochemistry.

Further benefits of leptin treatment throughout lactation in the offspring of gestational calorie-restricted dams were also observed when looking at circulating ghrelin levels. Ghrelin is an important hormone mainly secreted by the stomach that acts in the brain regulating food intake, body weight and adiposity, and glucose metabolism (Tschop et al., 2000). Here, unlike previous findings (Garcia et al., 2013), we did not observe a significant decrease in plasma ghrelin levels in CR animals compared to controls; nevertheless, we observed a significant increase in plasma ghrelin levels in CR animals that were treated with oral leptin throughout the suckling period (CR-Leptin animals) compared to CR animals, which was significant at the age of 3 months. The increased plasma ghrelin levels in CR-Leptin animals vs. CR animals seem to be related to increased gastric ghrelin secretion, in accordance with the increased number of TyrOH^+^ fibers in the stomach found at early ages. Nevertheless, it is worthy to mention that, although alterations in circulating ghrelin levels appear to be due to potential disturbances in the sympathetic innervation to the stomach, the influence of other factors cannot be ruled out.

The presence of increased ghrelin levels in CR-Leptin rats in adulthood, which are known to be protected from the increased susceptibility to develop obesity characteristic of maternal calorie restriction, could seem controversial when considering the orexigenic actions of ghrelin (Tschop et al., 2000; Kamegai et al., 2001). However, endogenous ghrelin does not appear to be essential in food intake regulation, as demonstrated in ghrelin-null mice, which do not exhibit appreciable alterations in the regulation of food intake or body weight (Wortley et al., 2004). Furthermore, plasma ghrelin levels have been reported to be reduced in animal models of obesity (Ariyasu et al., 2002; Levin et al., 2003) and in obese subjects, being inversely correlated with adiposity, fasting insulin, and leptin (Tschop et al., 2001). In this regard, we have found no differences in food intake between the different groups of animals, but CR animals are known to have a greater tendency toward excess fat accumulation along with higher insulin levels and HOMA-IR index, particularly when exposed to obesogenic diets (Palou et al., 2010; Szostaczuk et al., 2017). Therefore, the presence of increased ghrelin levels in CR-Leptin animals might account for the reversion of the above mentioned alterations, as previously described (Szostaczuk et al., 2017).

Leptin has a well-known role in the regulation of energy balance and is also considered to play a key role in the pathogenesis of obesity-related disorders. Besides the relevance of fasting levels of leptin and ghrelin individually, the L/G ratio may be more sensitive to predict alterations related with metabolic syndrome components, like obesity and insulin resistance (Hajimohammadi et al., 2017). A low L/G ratio has been considered a good marker to predict better metabolic adaptation and successful weight loss and maintenance after an energy-restricted diet (Crujeiras et al., 2010, 2014; Labayen et al., 2011). In this sense, we found here that adult CR animals displayed significantly greater values of the L/G ratio than controls, in accordance to the increased tendency of these animals to develop metabolic syndrome characteristics, particularly when exposed to an obesogenic diet (Szostaczuk et al., 2017). Notably, this ratio was completely normalized in CR animals treated with oral leptin throughout lactation. These findings, besides showing reversion of altered malprogramming structures and functions due to adverse prenatal conditions, are in favor of the potential usefulness of the L/G ratio measurement in adulthood as a clinical non-invasive marker of programmed susceptibility to develop obesity-related alterations; furthermore, it also seems to be able to detect the reversion of these disruptions by early life treatment, being more sensitive than leptin or ghrelin alone.

In summary, the present results shows for the first time that leptin supplementation, at physiological doses, during the lactation period to the offspring of gestational calorie-restricted dams reverses the decrease in TyrOH expression in the stomach associated with maternal under nutrition, which was particularly evident at early ages. Normalization of the sympathetic innervation in the stomach during lactation may contribute to the increase in circulating ghrelin levels and to the normalization of the L/G ratio in adulthood. These findings give further support to the benefits of the presence of leptin in breast milk compared to infant formulae, which lacks leptin.

## Author contributions

NS and JK carried out the experiments. NS, JS, AP, and CP designed the experiments and evaluated results. NS and JS wrote the first draft of the paper. CP and AP wrote the definitive version. All authors have revised the manuscript and approved the final version.

### Conflict of interest statement

AP, CP, and JS are authors of a patent held by the University of the Balearic Islands entitled “Use of leptin for the prevention of excess body weight and composition containing leptin” (WO 2006089987 A1) (Priority data: 23 February 2005). The other authors declare that the research was conducted in the absence of any commercial or financial relationships that could be construed as a potential conflict of interest.

## References

[B1] Ariyasu H. Takaya K. Hosoda H. Iwakura H. Ebihara K. Mori K.. (2002). Delayed short-term secretory regulation of ghrelin in obese animals: evidenced by a specific RIA for the active form of ghrelin. Endocrinology 143, 3341–3350. 10.1210/en.2002-220225 12193546

[B2] Casabiell X. Pineiro V. Tome M. A. Peino R. Dieguez C. Casanueva F. F. (1997). Presence of leptin in colostrum and/or breast milk from lactating mothers: a potential role in the regulation of neonatal food intake. J. Clin. Endocr. Metab. 82, 4270–4273. 10.1210/jcem.82.12.4590 9398752

[B3] Crujeiras A. B. Diaz-Lagares A. Abete I. Goyenechea E. Amil M. Martinez J. A.. (2014). Pre-treatment circulating leptin/ghrelin ratio as a non-invasive marker to identify patients likely to regain the lost weight after an energy restriction treatment. J. Endocrinol. Invest. 37, 119–126. 10.1007/s40618-013-0004-2 24497210

[B4] Crujeiras A. B. Goyenechea E. Abete I. Lage M. Carreira M. C. Martinez J. A.. (2010). Weight regain after a diet-induced loss is predicted by higher baseline leptin and lower ghrelin plasma levels. J. Clin. Endoc. Metab. 95, 5037–5044. 10.1210/jc.2009-2566 20719836

[B5] Garcia A. P. Palou M. Priego T. Sanchez J. Palou A. Pico C. (2010). Moderate caloric restriction during gestation results in lower arcuate nucleus NPY- and alphaMSH-neurons and impairs hypothalamic response to fed/fasting conditions in weaned rats. Diabetes Obes. Metab. 12, 403–413. 10.1111/j.1463-1326.2009.01174.x 20415688

[B6] Garcia A. P. Palou M. Sanchez J. Priego T. Palou A. Pico C. (2011). Moderate caloric restriction during gestation in rats alters adipose tissue sympathetic innervation and later adiposity in offspring. PLoS ONE 6:e17313. 10.1371/journal.pone.0017313 21364997 PMC3041800

[B7] Garcia A. P. Priego T. Palou M. Sanchez J. Palou A. Pico C. (2013). Early alterations in plasma ghrelin levels in offspring of calorie-restricted rats during gestation may be linked to lower sympathetic drive to the stomach. Peptides 39, 59–63. 10.1016/j.peptides.2012.11.005 23159561

[B8] Giordano A. Frontini A. Murano I. Tonello C. Marino M. A. Carruba M. O.. (2005). Regional-dependent increase of sympathetic innervation in rat white adipose tissue during prolonged fasting. J. Histochem. Cytochem. 53, 679–687. 10.1369/jhc.4A6566.2005 15928317

[B9] Gluckman P. D. Hanson M. A. Pinal C. (2005). The developmental origins of adult disease. Matern. Child Nutr. 1, 130–141. 10.1111/j.1740-8709.2005.00020.x 16881892 PMC6860944

[B10] Godfrey K. M. Barker D. J. (2001). Fetal programming and adult health. Public Health Nutr. 4, 611–624. 10.1079/PHN2001145 11683554

[B11] Hajimohammadi M. Shab-Bidar S. Neyestani T. R. (2017). Consumption of vitamin D-fortified yogurt drink increased leptin and ghrelin levels but reduced leptin to ghrelin ratio in type 2 diabetes patients: a single blind randomized controlled trial. Eur. J. Nutr. 56, 2029–2036. 10.1007/s00394-017-1397-z 28229278

[B12] Hosoda H. Kangawa K. (2008). The autonomic nervous system regulates gastric ghrelin secretion in rats. Regul. Pept. 146, 12–18. 10.1016/j.regpep.2007.07.005 17720259

[B13] Hsu S. M. Raine L. Fanger H. (1981). Use of avidin-biotin-peroxidase complex (ABC) in immunoperoxidase techniques: a comparison between ABC and unlabeled antibody (PAP) procedures. J. Histochem. Cytochem. 29, 577–580. 10.1177/29.4.6166661 6166661

[B14] Kamegai J. Tamura H. Shimizu T. Ishii S. Sugihara H. Wakabayashi I. (2001). Chronic central infusion of ghrelin increases hypothalamic neuropeptide Y and Agouti-related protein mRNA levels and body weight in rats. Diabetes 50, 2438–2443. 10.2337/diabetes.50.11.2438 11679419

[B15] Konieczna J. Garcia A. P. Sanchez J. Palou M. Palou A. Pico C. (2013). Oral leptin treatment in suckling rats ameliorates detrimental effects in hypothalamic structure and function caused by maternal caloric restriction during gestation. PLoS ONE 8:e81906. 10.1371/journal.pone.0081906 24312379 PMC3842976

[B16] Konieczna J. Palou M. Sanchez J. Pico C. Palou A. (2015). Leptin intake in suckling rats restores altered T3 levels and markers of adipose tissue sympathetic drive and function caused by gestational calorie restriction. Int. J. Obes. 39, 959–966. 10.1038/ijo.2015.22 25869480

[B17] Labayen I. Ortega F. B. Ruiz J. R. Lasa A. Simon E. Margareto J. (2011). Role of baseline leptin and ghrelin levels on body weight and fat mass changes after an energy-restricted diet intervention in obese women: effects on energy metabolism. J. Clin. Endocrinol. Metab. 96, E996–E1000. 10.1210/jc.2010-3006 21470990

[B18] Levin B. E. Dunn-Meynell A. A. Ricci M. R. Cummings D. E. (2003). Abnormalities of leptin and ghrelin regulation in obesity-prone juvenile rats. Am. J. Physiol. Endocrinol. Metab. 285, E949–E957. 10.1152/ajpendo.00186.2003 12865257

[B19] Livak K. J. Schmittgen T. D. (2001). Analysis of relative gene expression data using real-time quantitative PCR and the 2(-Delta Delta C(T)) method. Methods 25, 402–408. 10.1006/meth.2001.1262 11846609

[B20] Mundinger T. O. Cummings D. E. Taborsky G. J. Jr. (2006). Direct stimulation of ghrelin secretion by sympathetic nerves. Endocrinology 147, 2893–2901. 10.1210/en.2005-1182 16527847

[B21] Palou A. Pico C. (2009). Leptin intake during lactation prevents obesity and affects food intake and food preferences in later life. Appetite 52, 249–252. 10.1016/j.appet.2008.09.013 18926866

[B22] Palou M. Konieczna J. Torrens J. M. Sanchez J. Priego T. Fernandes M. L.. (2012). Impaired insulin and leptin sensitivity in the offspring of moderate caloric-restricted dams during gestation is early programmed. J. Nutr. Biochem. 23, 1627–1639. 10.1016/j.jnutbio.2011.11.005 22444870

[B23] Palou M. Priego T. Romero M. Szostaczuk N. Konieczna J. Cabrer C.. (2015). Moderate calorie restriction during gestation programs offspring for lower BAT thermogenic capacity driven by thyroid and sympathetic signaling. Int. J. Obes. 39, 339–345. 10.1038/ijo.2014.56 24694665

[B24] Palou M. Priego T. Sanchez J. Palou A. Pico C. (2010). Sexual dimorphism in the lasting effects of moderate caloric restriction during gestation on energy homeostasis in rats is related with fetal programming of insulin and leptin resistance. Nutr. Metab. 7:69. 10.1186/1743-7075-7-69 20796266 PMC2939651

[B25] Pico C. Oliver P. Sanchez J. Miralles O. Caimari A. Priego T.. (2007). The intake of physiological doses of leptin during lactation in rats prevents obesity in later life. Int. J. Obes. 31, 1199–1209. 10.1038/sj.ijo.0803585 17356529

[B26] Picó C. Palou M. Priego T. Sánchez J. Palou A. (2012). Metabolic programming of obesity by energy restriction during the perinatal period: different outcomes depending on gender and period, type and severity of restriction. Front. Physiol. 3:436. 10.3389/fphys.2012.00436 23189059 PMC3504314

[B27] Poykko S. M. Kellokoski E. Horkko S. Kauma H. Kesaniemi Y. A. Ukkola O. (2003). Low plasma ghrelin is associated with insulin resistance, hypertension, and the prevalence of type 2 diabetes. Diabetes 52, 2546–2553. 10.2337/diabetes.52.10.2546 14514639

[B28] Ravelli G. P. Stein Z. A. Susser M. W. (1976). Obesity in young men after famine exposure *in utero* and early infancy. N. Engl. J. Med. 295, 349–353. 10.1056/NEJM197608122950701 934222

[B29] Sanchez J. Oliver P. Miralles O. Ceresi E. Pico C. Palou A. (2005). Leptin orally supplied to neonate rats is directly uptaken by the immature stomach and may regulate short-term feeding. Endocrinology 146, 2575–2582. 10.1210/en.2005-0112 15746250

[B30] Sanchez J. Oliver P. Palou A. Pico C. (2004). The inhibition of gastric ghrelin production by food intake in rats is dependent on the type of macronutrient. Endocrinology 145, 5049–5055. 10.1210/en.2004-0493 15284203

[B31] Sanchez J. Priego T. Palou M. Tobaruela A. Palou A. Pico C. (2008). Oral supplementation with physiological doses of leptin during lactation in rats improves insulin sensitivity and affects food preferences later in life. Endocrinology 149, 733–740. 10.1210/en.2007-0630 17991728

[B32] Spencer S. J. Emmerzaal T. L. Kozicz T. Andrews Z. B. (2015). Ghrelin's role in the hypothalamic-pituitary-adrenal axis stress response: implications for mood disorders. Biol. Psychiatry 78, 19–27. 10.1016/j.biopsych.2014.10.021 25534754

[B33] Szostaczuk N. Priego T. Palou M. Palou A. Pico C. (2017). Oral leptin supplementation throughout lactation in rats prevents later metabolic alterations caused by gestational calorie restriction. Int. J. Obes. 41, 360–371. 10.1038/ijo.2016.241 28028317

[B34] Tschop M. Smiley D. L. Heiman M. L. (2000). Ghrelin induces adiposity in rodents. Nature 407, 908–913. 10.1038/35038090 11057670

[B35] Tschop M. Weyer C. Tataranni P. A. Devanarayan V. Ravussin E. Heiman M. L. (2001). Circulating ghrelin levels are decreased in human obesity. Diabetes 50, 707–709. 10.2337/diabetes.50.4.707 11289032

[B36] Vickers M. H. Breier B. H. Cutfield W. S. Hofman P. L. Gluckman P. D. (2000). Fetal origins of hyperphagia, obesity, and hypertension and postnatal amplification by hypercaloric nutrition. Am. J. Physiol. Endocrinol. Metab. 279, E83–E87. 10.1152/ajpendo.2000.279.1.E83 10893326

[B37] Wortley K. E. Anderson K. D. Garcia K. Murray J. D. Malinova L. Liu R. (2004). Genetic deletion of ghrelin does not decrease food intake but influences metabolic fuel preference. Proc. Natl. Acad. Sci. U.S.A. 101, 8227–8232. 10.1073/pnas.0402763101 15148384 PMC419585

[B38] Wren A. M. Seal L. J. Cohen M. A. Brynes A. E. Frost G. S. Murphy K. G.. (2001). Ghrelin enhances appetite and increases food intake in humans. J. Clin. Endocrinol. Metab. 86:5992. 10.1210/jcem.86.12.8111 11739476

