# Supplementary material for: Leptin Intake at Physiological Doses Throughout Lactation in Male Wistar Rats Normalizes the Decreased Density of Tyrosine Hydroxylase-Immunoreactive Fibers in the Stomach Caused by Mild Gestational Calorie Restriction
Source: Front Physiol. 2018 Mar 21;9:256. doi: 10.3389/fphys.2018.00256 (PMC5871795; doi:10.3389/fphys.2018.00256)
Supplement: Supplementary file 1 [file Image1.PDF]

Supplementary Material

Leptin intake at physiological doses throughout lactation in male Wistar rats normalizes the decreased density of tyrosine hydroxylase-immunoreactive fibers in the stomach caused by mild gestational calorie restriction

Nara Szostaczuk, Juana Sánchez, Jadwiga Konieczna, Andreu Palou*, Catalina Picó.

***Correspondence:**
Prof. Andreu Palou.
andreu.palou@uib.es

SUPPLEMENTARY FIGURES

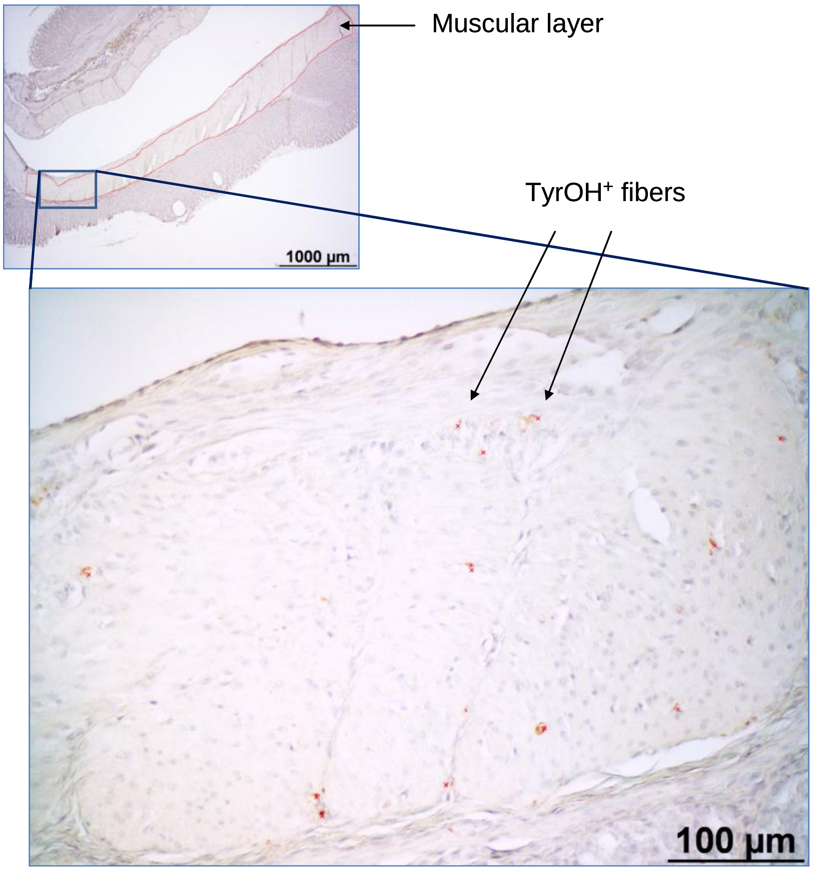

Figure S1. Picture illustrating how the counting of TyrOH positive (TyrOH$^+$) fibers in the muscular layer of the stomach was made. The area measured is indicated in red color. Counting was made from the left side to the right side. TyrOH$^+$ fibers counted in the inset are marked with a red cross. The image corresponds to a 25-day-old control animal.

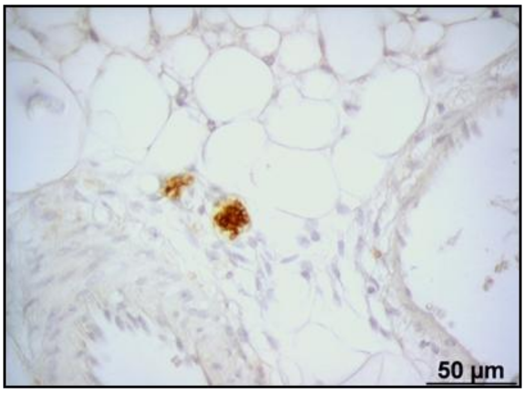

Figure S2. Picture showing representative TyrOH positive (TyrOH$^+$) nerves in the parenchyma of the stomach, which were used as internal positive controls. The nerves contain abundant TyrOH$^+$ fibers. The image corresponds to a 25-day-old control animal.
